# Development and validation of a cardiovascular risk prediction model in type 1 diabetes

**DOI:** 10.1007/s00125-021-05478-4

**Published:** 2021-06-09

**Authors:** Stuart J. McGurnaghan, Paul M. McKeigue, Stephanie H. Read, Stefan Franzen, Ann-Marie Svensson, Marco Colombo, Shona Livingstone, Bassam Farran, Thomas M. Caparrotta, Luke A. K. Blackbourn, Joseph Mellor, Ioanna Thoma, Naveed Sattar, Sarah H. Wild, Soffia Gudbjörnsdottir, Helen M. Colhoun, on behalf of the Swedish National Diabetes Register and the Scottish Diabetes Research Network Epidemiology Group

**Affiliations:** 1 Institute of Genetics and Cancer, University of Edinburgh, Edinburgh, UK; 2 The Usher Institute, University of Edinburgh, Edinburgh, UK; 3 Women’s College Research Institute, Women’s College Hospital, Toronto, ON Canada; 4 Department of Medicine, Sahlgrenska University Hospital, University of Gothenburg, Gothenburg, Sweden; 5 Diabetes Epidemiology Unit, University of Dundee, Dundee, UK; 6 BHF Glasgow Cardiovascular Research Centre, University of Glasgow, Glasgow, UK; 7 Department of Public Health, NHS Fife, Kirkcaldy, UK

**Keywords:** Cardiovascular, Risk prediction, Type 1 diabetes

## Abstract

**Aims/hypothesis:**

We aimed to report current rates of CVD in type 1 diabetes and to develop a CVD risk prediction tool for type 1 diabetes.

**Methods:**

A cohort of 27,527 people with type 1 diabetes without prior CVD was derived from the national register in Scotland. Incident CVD events during 199,552 person-years of follow-up were ascertained using hospital admissions and death registers. A Poisson regression model of CVD was developed and then validated in the Swedish National Diabetes Register (*n* = 33,183). We compared the percentage with a high 10 year CVD risk (i.e., ≥10%) using the model with the percentage eligible for statins using current guidelines by age.

**Results:**

The age-standardised rate of CVD per 100,000 person-years was 4070 and 3429 in men and women, respectively, with type 1 diabetes in Scotland, and 4014 and 3956 in men and women in Sweden. The final model was well calibrated (Hosmer–Lemeshow test *p* > 0.05) and included a further 22 terms over a base model of age, sex and diabetes duration (C statistic 0.82; 95% CI 0.81, 0.83). The model increased the base model C statistic from 0.66 to 0.80, from 0.60 to 0.75 and from 0.62 to 0.68 in those aged <40, 40–59 and *≥* 60 years, respectively (all *p* values <0.005). The model required minimal calibration in Sweden and had a C statistic of 0.85. Under current guidelines, >90% of those aged 20–39 years and 100% of those *≥*40 years with type 1 diabetes were eligible for statins, but it was not until age 65 upwards that 100% had a modelled risk of CVD *≥*10% in 10 years.

**Conclusions/interpretation:**

A prediction tool such as that developed here can provide individualised risk predictions. This 10 year CVD risk prediction tool could facilitate patient discussions regarding appropriate statin prescribing. Apart from 10 year risk, such discussions may also consider longer-term CVD risk, the potential for greater benefits from early vs later statin intervention, the potential impact on quality of life of an early CVD event and evidence on safety, all of which could influence treatment decisions, particularly in younger people with type 1 diabetes.

**Graphical abstract:**

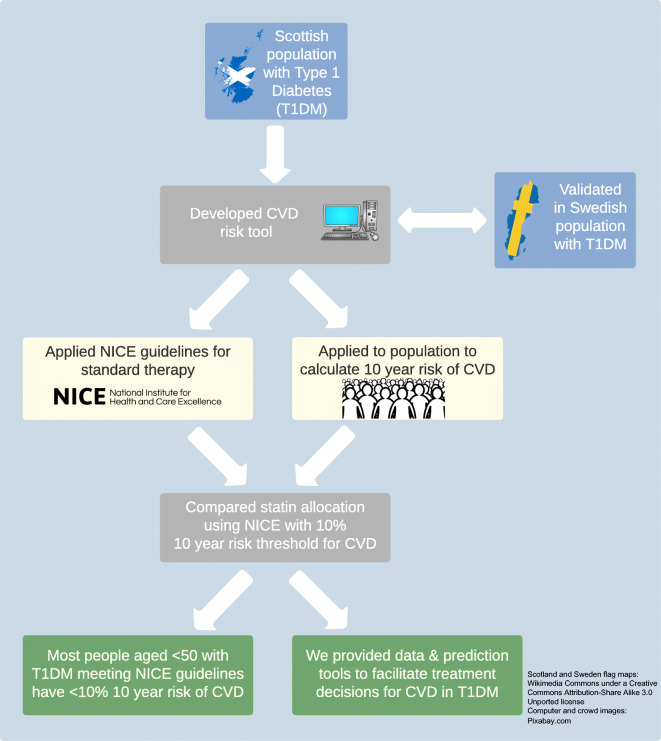

**Supplementary Information:**

The online version contains peer-reviewed but unedited supplementary material available at 10.1007/s00125-021-05478-4.

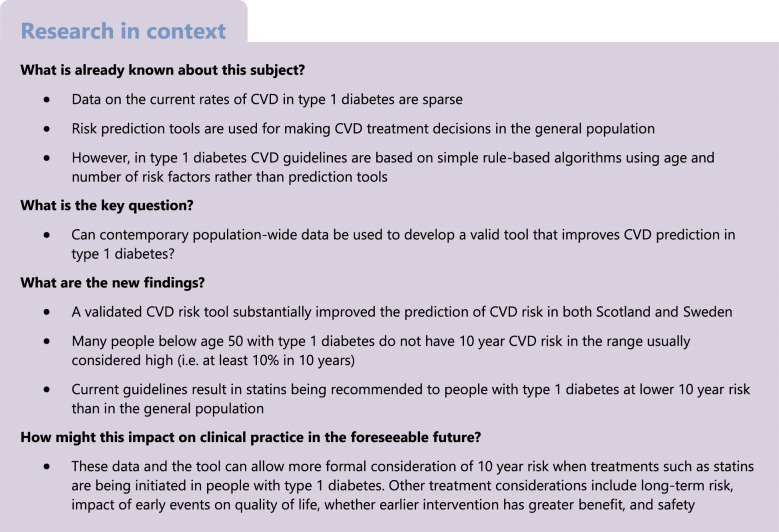



## Introduction

In people with type 1 diabetes CVD is a major cause of loss of life expectancy [1] and CVD risk is elevated two- to fourfold [2, 3]. CVD risk assessment tools can aid the identification of individuals who would benefit from preventive treatments, for guiding decisions on intensification of therapies, and can improve risk communication to patients. In the general population, treatment guidelines allocate treatment to those reaching various risk thresholds using such tools. For example, in the UK the National Institute for Health and Care Excellence (NICE) recommends using the QRISK2 or QRISK3 (ClinRisk, UK) risk score [4, 5] to assign statin treatment to those with ≥10% 10 year risk of CVD [6]. For those with type 1 diabetes, however, crude rule-based algorithms are used [6, 7]. For example, the UK NICE guidelines recommend statins in all those with type 1 diabetes aged ≥40 years or those ≥20 years with at least one additional CVD risk factor [6]. The most recent ESC/EASD guidelines designate all with type 1 diabetes as being at ‘high risk’ of CVD and warranting statin with the exception of those below age 35 years and with diabetes duration <10 years and without other risk factors [8]. Since data on contemporaneous risks of CVD in type 1 diabetes are lacking, it is not clear what risk threshold current guidelines are implicitly using in type 1 diabetes. Furthermore, such rule-based algorithms ignore substantial variation in risk that might be predictable; accordingly, the development of a CVD risk score in type 1 diabetes has been called for [7].

Although there are two published tools that might be used [5, 9], they have not been widely adopted or promoted in the most recent guidelines [8]. This may be because neither has had international validation [5, 9], or because quantification of how such tools assign risks compared with rule-based algorithms is needed to convincingly demonstrate their utility.

Therefore, using data from the Scottish and Swedish national diabetes registers, we aimed to: (1) provide contemporary data on rates of CVD in people with type 1 diabetes; (2) develop and internationally validate a CVD risk prediction model for first CVD events in people with type 1 diabetes; (3) compare its performance with the two tools previously reported for first events; and (4) compare the percentage with various thresholds for 10 year CVD risk using the model with the percentage eligible for statins using the NICE guidelines.

## Methods

### Study design and data sources

#### Scottish cohort

The cohort was formed using a 2019 extract from the previously described Scottish Care Information – Diabetes (SCI-Diabetes) dataset [2]. Briefly, since 2004 SCI-Diabetes has collated demographic and clinical data, including issued prescriptions and retinopathy screening data, for over 99% of people nationally with an assigned diagnosis of diabetes. The cohort for this study consisted of adults aged 18 years and above who were diagnosed with type 1 diabetes below 50 years. Those with a previous CVD event were omitted. The study started on 1 January 2008 and ended on 1 January 2018. Each person’s entry date was defined as the latest of study start date, date of diabetes diagnosis, date of 18th birthday or date of first coming under observation with diabetes in the national register. The exit date was defined as the earliest of study end date, date of death, date of incident CVD event or ceasing to be under observation in the national register. CVD outcome data were acquired through linkage to the Scottish Morbidity Records, the National Health Service admissions dataset and the death registrations held by the General Register Office for Scotland. CVD was defined as any hospital admission or death due to myocardial infarction, stroke, unstable angina, transient ischaemic attack or peripheral vascular disease; or any coronary, carotid or peripheral artery revascularisations; or major amputation procedures; or any death due to these conditions; or acute coronary heart disease. See the electronic supplementary material (ESM) Methods for the International Classification of Disease version 10 codes and Office of Population Censuses and Surveys Classification of Interventions and Procedures (OPCS-4) codes within this definition.

#### Candidate covariates

Factors reported in the literature as predicting CVD risk in type 1 diabetes or included in previous risk models for CVD were included as covariates for selection [4, 10] and are listed in ESM Table 1. These were derived from SCI-Diabetes and linked datasets including prescribing data. Baseline measurements were those nearest to and prior to the time of study entry but no more than 24 months before study entry. We included a covariate for the mean HbA_1c_ in the preceding 3 years. Measurements were defined at baseline apart from current age which was time-updated at the beginning of each person-time interval. Covariates with 60% or more missingness were excluded from the analyses. See the ESM Methods for further details on covariate definition.

#### External validation cohort

The external validation cohort was from the Swedish National Diabetes Register (NDR), which has nationwide coverage, and to which trained clinicians report clinical information [10]. This registry was initiated in 1996 and includes data for almost all people with type 1 diabetes aged over 18 years. The study start and end dates for this cohort were 1 January 2002 and 31 December 2013, respectively. CVD event data were obtained by linkage to the Swedish Inpatient Register and Cause of Death registries. Risk factors were defined as for Scotland, except that deprivation was measured using quintiles of disposable income.

### Statistical analyses

Age-standardised incidence rates for CVD by sex were estimated by direct standardisation to the 2013 European Standard Population [11]. Missing covariate data in both cohorts were imputed using multiple imputation using an expectation–maximisation with bootstrapping (EMB) algorithm, assuming data were missing at random and agnostic to the CVD outcome, implemented in the *Amelia II* package in R [12, 13]. Ten multiply imputed datasets were created and for continuous variables the mean of the imputed values was used in the regression model. Categorical variables were converted to probabilities for each category, reflecting the frequency of each category across the ten imputations.

For model derivation, within the Scottish cohort, person-time was split into 1 year intervals, with each observation constituting a 1 year person-time interval. A base model including the baseline and time-updated age, sex and baseline diabetes duration and an offset term for intervals in which censoring occurred was generated first using Poisson regression using the R package *glm*. Forward selection was then used to add risk factors to the model as long as the Akaike information criterion fell by at least the number of extra parameters (i.e., with the *k* = 3 setting in the R package *step*). Covariates with a skewed distribution (eGFR, total cholesterol:HDL-cholesterol ratio and BMI) were log transformed. Quadratic and cubic terms were entered for age, since we know a priori that the relationship with CVD is likely non-linear. Interactions between each candidate covariate and age and sex were considered for inclusion in the model. The cumulative survival free of CVD by any given attained age, conditional on not otherwise being censored, was generated using the current age- and sex-specific average survival probabilities generated by the Poisson model. Survival probabilities were obtained using the *predict* function in R. See the ESM Methods for model sensitivity checks.

Predictive performance was examined in a 20-fold training test cross-validation framework. The dataset was partitioned into 20 disjoint test sets each comprising 5% of the study population. For each test set the remaining 95% of the population constituted the training set. The modelling process was then run on each training set and the resultant model was applied to its test set, thus yielding predicted values for that test set. The 20 test sets each with an observed and predicted value for each individual in that test set were concatenated into a single dataset that was used to evaluate predictive performance. Using this combined test dataset, the increment in discrimination achieved with the final model compared with the base model was quantified using the C statistic and the expected information for discrimination, Λ, which is expressed in bits [14]. Increments in Λ are interpretable in absolute units whereas increments in C statistic are not. Λ was computed with the R package *wevid* [15]. The *pROC* package was used to compute the C statistic as the area under the receiver operating characteristic (ROC) curve, using the trapezoidal rule equivalent to Harrell’s C statistic. The strength of evidence that the final model improved the predictive performance on top of the base model was assessed by the increment in test log-likelihood; a difference in test log-likelihood of 6.9 natural log units is asymptotically equivalent to a *p* value less than 0.005 for comparison of nested models [16]. Calibration was assessed visually using calibration plots and by testing whether the observed and predicted counts of events differed significantly across deciles of risk using the Hosmer–Lemeshow test. See the ESM Methods for special considerations on evaluating predictive performance from survival models that informed the evaluation. For external validation in the Swedish cohort, the model was first re-calibrated by setting the intercept so as to equate the total observed and predicted events, since we expect a priori that incidence rates will differ between countries, and the predictive performance was then evaluated as above (see the ESM Methods for further details).

In the Scottish cohort we calculated the percentage of individuals in Scotland who would receive statins under the NICE guidelines, i.e., those with age ≥ 40 years or having diabetes for more than 10 years or having established nephropathy or at least one other CVD-related risk factor by age band. Using the model, we calculated the percentage in each age band in Scotland that had a 10 year predicted risk of CVD of ≥10% and also of ≥5%. For the purpose of this calculation, the predicted risk of all those on a statin already at baseline was increased by 25% to account crudely for the typical reduction in risk expected with statin therapy.

We applied risk equations from the Steno CVD Type 1 Engine and the QRISK3 model to the Scottish dataset and compared the resultant expected with observed events and calculated the C statistic using the *pROC* package.

## Results

### Age-standardised rates of CVD in Scotland and Sweden

Overall, 27,527 individuals with type 1 diabetes were included in the final cohort in Scotland following the exclusion of 1952 individuals with a previous history of CVD. There were 2790 CVD events during 199,552 person-years of follow-up. Of first events, 51% were coronary, 11% cerebrovascular or carotid, and 38% peripheral vascular. Baseline characteristics of those developing vs not developing CVD are shown in ESM Table 1.

The Swedish cohort included 33,183 people in whom there were 3262 incident CVD events during 253,197 person-years of follow-up. The confidence intervals for the sex-specific age-standardised rates overlapped in Scotland and Sweden and rates were <600/100,000 person-years at age 18–39 in both (Table 1).

**Table 1:** Age-standardised CVD rates in Scotland and Sweden

Sex	Age	*N* events		Follow-up (person-years)		Age-standardised rate (95% CI)	
		Scotland	Sweden	Scotland	Sweden	Scotland	Sweden
Male	18–39	379	349	65,795.59	92,567.20	599 (540, 662)	472 (432, 514)
	40–59	937	1047	42,013.01	38,756.81	2491 (2324, 2668)	3015 (2830, 3209)
	60–95	342	405	5,612.36	5664.05	9995 (7320, 14,041)	9478 (7520, 12,909)
Female	18–39	281	272	49,971.22	76,465.07	598 (530, 673)	372 (329, 420)
	40–59	564	772	30,681.44	33,738.62	1995 (1826, 2178)	2435 (2263, 2617)
	60–95	287	417	5478.82	6005.46	8417 (6370, 11,263)	9952 (7980, 12,846)
Male	All	1658	1801	113,420.95	136,988.06	4070 (3240, 5285)	4014 (3406, 5046)
Female	All	1132	1461	86,131.48	116,209.15	3429 (2793, 4285)	3956 (3346, 4829)

Event rates are per 100,000 person-years, age standardised to the 2013 European Standard Population

### Model development and performance in the Scottish dataset

**Fig. 1:**
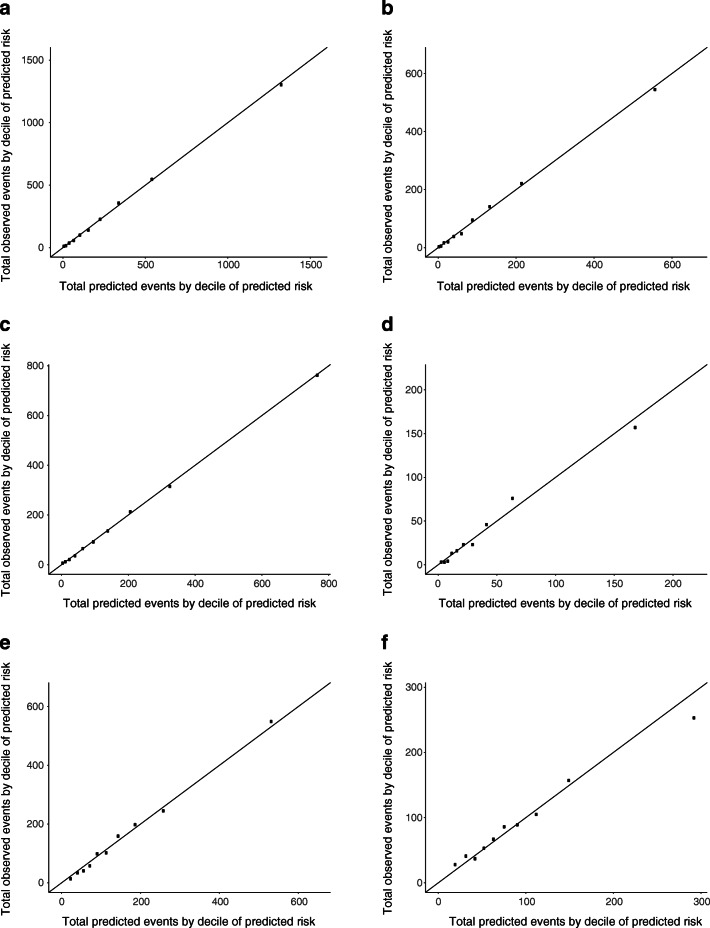
(**a**–**f**) Calibration of the model in the Scottish cross-validation test dataset overall and by sex and age band. (**a**) All (199,552 person-years). (**b**) Female (86,131 person-years). (**c**) Male (113,421 person-years). (**d**) Age at entry 20–39 years (89,089 person-years). (**e**) Age at entry 40–59 years (73,005 person-years). (**f**) Age at entry ≥60 years (11,197 person-years)

Most covariates had less than 20% missingness (ESM Table 2). For most of the risk factors of interest adjusted for age, sex and diabetes duration there were statistically significant associations with CVD (ESM Table 3). Using forward selection, the final model included 22 risk factor terms, including quadratic and cubic terms for current age and interaction terms (Table 2). The final model increased the model C statistic compared with the baseline model in all age–sex strata (Table 3). Using Λ, the improvement in predictive performance was greatest in the youngest age band (Table 3). The increment in test log-likelihood of the model compared with baseline was sufficiently large in all age–sex strata to be statistically significant (*p* < 0.005). The model was well calibrated, with a Hosmer–Lemeshow test result that was non-significant at *p* = 0.7 (Fig. 1).

**Table 2:** Final multivariable Poisson model regression results

Predictor	IRR	(95% CI)	*p* value
Age at entry (years)	0.967	(0.954, 0.980)	<0.001
Current age (years)	1.384	(1.279, 1.497)	<0.001
Current age^2^ (years^2^)	0.996	(0.995, 0.998)	<0.001
Current age^3^ (years^3^)	1.000	(1.000, 1.000)	<0.001
Sex			
Male	1 (reference)		
Female	0.402	(0.277, 0.583)	<0.001
Diabetes duration (years at entry)	1.020	(1.017, 1.024)	<0.001
Deprivation index			
Quintile 1 (most deprived)	1 (reference)		
Quintile 2	0.893	(0.800, 0.996)	0.043
Quintile 3	0.751	(0.667, 0.845)	<0.001
Quintile 4	0.680	(0.600, 0.771)	<0.001
Quintile 5 (least deprived)	0.572	(0.499, 0.657)	<0.001
HbA_1c_ (mmol/mol)	1.011	(1.001, 1.021)	0.029
Mean HbA_1c_ (last 3 years) (mmol/mol)	1.027	(1.015, 1.040)	<0.001
log BMI (kg/m^2^)	0.146	(0.058, 0.368)	<0.001
Height (m)	0.058	(0.019, 0.178)	<0.001
Weight (kg)	1.008	(0.993, 1.024)	0.304
Systolic BP (mmHg)	1.004	(1.002, 1.007)	0.001
log total cholesterol:HDL-cholesterol ratio (mmol/l)	1.641	(1.405, 1.917)	<0.001
log eGFR (ml min^−1^ 1.73 m^−2^)	0.706	(0.595, 0.837)	<0.001
Albuminuric grade			
Normal	1 (reference)		
Macro	2.505	(2.129, 2.948)	<0.001
Micro	1.407	(1.268, 1.561)	<0.001
Retinopathy grading			
No retinopathy	1 (reference)		
Non-referable	1.103	(0.982, 1.239)	0.097
Referable or eye clinic	1.502	(1.341, 1.683)	<0.001
Tobacco smoking			
Never smoked	1 (reference)		
Ever smoked	1.405	(1.289, 1.531)	<0.001
Treated for hypertension	1.357	(1.236, 1.489)	<0.001
Treated for dyslipidaemia	1.150	(1.045, 1.267)	0.004
Ever atrial fibrillation	1.857	(1.181, 2.919)	0.007
Interaction: Age × 3 year mean HbA_1c_	1.000	(1.000, 1.000)	<0.001
Interaction: Age × weight	1.000	(1.000, 1.000)	0.016
Interaction: Sex × HbA_1c_	1.003	(1.000, 1.007)	0.079
Interaction: Sex × log total cholesterol:HDL-cholesterol ratio	1.481	(1.168, 1.879)	0.001

Normal albuminuria is an albumin/creatinine ratio < 30, micro is 30–300 and macro is >300 mg/l

IRR, incidence rate ratio

**Table 3:** Increment in the prediction of incident CVD from the base model to the final model in Scottish and Swedish validation datasets^a^

Group	C statistic (base) (95% CI)	C statistic (final) (95% CI)	Λ (base)	Λ (final)	log-likelihood increment (natural log units)
Scotland					
All	0.75 (0.74, 0.76)	0.82 (0.81, 0.83)	0.62	1.22	868.43
Male	0.76 (0.75, 0.77)	0.81 (0.80, 0.82)	0.61	1.18	439.33
Female	0.74 (0.73, 0.76)	0.83 (0.82, 0.84)	0.61	1.26	429.10
20–39	0.66 (0.64, 0.69)	0.80 (0.78, 0.82)	0.16	0.97	201.59
40–59	0.60 (0.59, 0.62)	0.75 (0.74, 0.76)	0.11	0.60	533.91
60+	0.62 (0.60, 0.64)	0.68 (0.67, 0.70)	0.15	0.38	125.63
Sweden					
All	0.80 (0.80, 0.81)	0.85 (0.85, 0.86)	0.91	1.64	211.10
Male	0.80 (0.80, 0.82)	0.85 (0.84, 0.86)	0.90	1.64	71.57
Female	0.80 (0.80, 0.82)	0.85 (0.85, 0.86)	0.91	1.65	515.27
20–39	0.72 (0.70, 0.75)	0.82 (0.79, 0.84)	0.48	1.17	802.65
40–59	0.66 (0.65, 0.67)	0.75 (0.74, 0.76)	0.23	0.70	434.05
60+	0.61 (0.60, 0.63)	0.66 (0.65, 0.67)	0.11	0.32	368.60

An increment in the test log-likelihood ≥6.9 natural log units is asymptotically equivalent to a *p* value <0.005, therefore all results are highly significant

Information for discrimination Λ is measured in bits

^a^Base is the base model adjusted for age, sex and duration. Final is the final model including all covariates selected by forward selection and detailed in the Poisson regression output

### External validation in the Swedish cohort

See ESM Table 4 for characteristics, ESM Table 5 for missingness and ESM Table 6 for risk factor associations in Sweden. To recalibrate the model, a recalibration factor γ of 0.06 was required to achieve calibration (ESM Fig. 1). This effectively reflects a 6% higher average risk in the Swedish data. As shown in Table 3, the increment in test log-likelihood shows that the model significantly improved prediction of CVD in Sweden in all age bands and in both sexes (all differences were at least 20 natural log units, *p* < 0.005) beyond the base model. The overall C statistic increased from 0.80 for the base model to 0.85 for the final model. The increment in prediction was greatest in the youngest age band (increment in Λ of 0.69 bits of information) and was similar in both sexes.

### Use of the model and web application

The final model intercept and coefficients are given in Table 4. Instructions for use are given in the ESM Results. A web-based Shiny application implementing the final model is available at: https://diabepi.shinyapps.io/cvdrisk/. This is for illustrative purposes only as a tool for clinical use would require device approval before use in many countries.

**Table 4:** Predictor variables and coefficients for risk prediction of CVD in type 1 diabetes

Predictor	Coefficient
(Intercept)	−4.03403744
Current age (years)	0.32483759
Current age^2^ (years^2^)	−0.00389356
Current age^3^ (years^3^)	1.993 × 10^−5^
Sex male	0.00000000
Sex female	−0.91153788
Diabetes duration (years)	0.0201993
Deprivation quintile 1 (most deprived)	0.00000000
Deprivation quintile 2	−0.11366821
Deprivation quintile 3	−0.28641292
Deprivation quintile 4	−0.38497544
Deprivation quintile 5 (least deprived)	−0.55777639
HbA_1c_ (mmol/mol)	0.01071311
Mean HbA_1c_ (last 3 years) (mmol/mol)	0.02712284
log BMI (kg/m^2^)	−1.9247447
Height (m)	−2.84393889
Weight (kg)	0.00802065
Systolic BP (mmHg)	0.0041725
log total cholesterol:HDL-cholesterol ratio (mmol/l)	0.49545924
log eGFR (ml min^−1^ 1.73 m^−2^)	−0.34828245
Albuminuric grade normal	0.00000000
Albuminuric grade micro	0.34128044
Albuminuric grade macro	0.91835596
Retinopathy none	0.00000000
Retinopathy non-referable	0.09832872
Retinopathy referable or eye clinic	0.40705123
Never smoked	0.00000000
Tobacco smoking	0.34002647
Treated for hypertension	0.30504081
Treated for dyslipidaemia	0.14012891
Ever atrial fibrillation	0.61882651
Interaction: Sex × log total cholesterol:HDL-cholesterol ratio	0.39282042
Interaction: Age × weight	0.00023665
Interaction: Sex × HbA_1c_	0.00335224

### Comparison of performance with previously published risk scores

When applied to the Scottish dataset, discriminative performance of the Steno model was similar and the QRISK3 much lower than for the model we developed in Scotland (ESM Table 7). The Steno model substantially overestimated predicted events and the QRISK3 model substantially underpredicted events in Scotland.

### Proportions eligible for statin therapy using NICE guidelines and the proportions exceeding 10% and 5% 10 year risk thresholds

If the NICE 2016 guidelines were applied in practice, then 100% of those with type 1 diabetes without prior CVD would be prescribed statin from age 40 years, with 81–90% being assigned statin from age bands in the 20–40 year range (ESM Table 8). However, the percentage with a 10 year risk ≥10% does not near 100% until age band 65–70 years, even allowing for statin effects on risks. The percentage with a 10 year risk of CVD ≥5% reaches 100% by age 55 years (ESM Table 8).

### Cumulative survival free of CVD

**Fig. 2:**
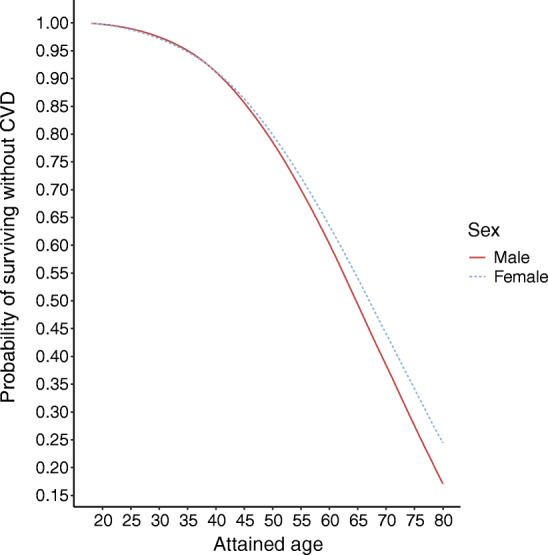
Probability of surviving without CVD, conditional on not being censored by non-CVD death, based on age- and sex-specific averaged survival probabilities from the model

Figure 2 shows that about 80% of women and men would be expected to remain free of CVD by age 50, but just 50% remain free of CVD by age 65 in men and 67.5 in women if current age-specific risks are applied across the life course.

### Alternative model without a socioeconomic indicator

For countries without a socioeconomic indicator, using the same process we produced an alternative model omitting Scottish Index of Multiple Deprivation (SIMD) from the forward selection (ESM Table 9) which had a slightly lower performance (Λ of 1.19 vs 1.22).

## Discussion

### Current rates of CVD in type 1 diabetes

We have shown broadly similar current age-standardised incidence rates of CVD between the Scottish and Swedish datasets for the group of events studied, with low rates in those below age 40 in both countries. The cumulative survival curve shows that at current disease rates 80% remain free of CVD by age 50 years. Thus, current guidelines that label most younger people with type 1 diabetes as being a high-risk group could create an erroneous impression of the risk of CVD unless the timeframe is appropriately specified. On the other hand, it can be seen that incidence rises steeply with age such that by their mid-sixties about half of those with type 1 diabetes will have developed CVD.

This study is by far the largest recent study of CVD rates in type 1 diabetes and the first to show rates for two whole countries. Direct comparison with other smaller studies is made difficult because of the varying specifications of clinical events and the uncertainty in representativeness of clinic-based populations for the background population being studied. In the Steno Diabetes Centre study for the years 2001–2013, the crude overall reported rate was much higher than ours at 27/1000 person-years [9], but, for the same period, the validation cohort from a different part of Denmark had an event rate much nearer ours at 17/1000 person-years. The Pittsburgh Epidemiology Data Center (EDC) reported higher CVD rates than in our cohort, though the wide confidence intervals in that study overlap ours in these age bands [17]. A previous analysis of primary care records in the UK described lower incidence rates than here [18], but did not include unstable angina or peripheral vascular disease.

### Risk prediction model

We have developed and internally validated a well-calibrated risk prediction model with good discriminative performance. We have shown that in Sweden the model was good at discrimination and little recalibration was required. Our development and validation cohorts were large, with altogether 7767 CVD events. Previously, a tool was developed with just 95 events [19] in the EURODIAB Prospective Complications Study, and another from Sweden included both primary and secondary events (*n* = 197) [20], and neither of these has been widely adopted. The QRISK3 model developed in the general primary care population in England had poor predictive performance in our data partly because it contains many variables that are only captured routinely in settings using the primary care software used in its development cohort. Here, we have provided international validation with the Steno Diabetes Centre score based on a much smaller cohort with 793 events [9]. It showed similar discrimination in our population to our own model, but substantial recalibration would be needed to apply it in our population or in Sweden as it overestimated risks by about 27%.

### Potential utility of the model in clinical decision making

We have shown that current algorithmic approaches for statin therapy initiation in type 1 diabetes using the NICE guidelines assign everyone aged 40 and over, and more than 90% of those aged 20 and over, to statin therapy. The recent European Societies’ guidelines assign more to statin therapy than the NICE guideline and label most adults with type 1 diabetes as being at ‘high risk’ for CVD. For the general population NICE uses a 10 year risk threshold for allocating statins, but the majority of those younger people with type 1 diabetes eligible for statins using the NICE guideline do not have risks of this level. Our purpose in making this comparison is not to advocate for altering guidelines to reduce eligibility for statins in type 1 diabetes. Rather, it is to stimulate discussion about the reasoning behind different guidelines and to enable future guidelines to be informed by current rather than out-of-date assumptions about absolute risks. Guideline committees, clinicians and people with diabetes might consider that for a given absolute 10 year risk level there is a rationale for being more assertive in introducing preventive therapies in type 1 diabetes than in the general population. If so, the rationale should be made explicit.

Arguments in favour of more assertive therapy at a given absolute 10 year risk in type 1 diabetes might include considerations of lifetime risk and of the loss of quality-adjusted life years from very early CVD events. Other justifications for introducing lipid-lowering therapy earlier in life and at lower absolute 10 year risk thresholds have been elegantly argued elsewhere [21]. The arguments include the dramatic effects of genetic mutations that lower LDL-cholesterol from early life on CVD risks, meta-analyses that found lower relative risk reductions at older than younger ages with statins and the lack of any evidence of diminution in effect of statins with longer-term use. Also relevant is that cost effectiveness of statin therapy at population level down to a 5% 10 year risk has been demonstrated [22], as well as the excellent safety profile of statin therapy. These arguments have to be balanced against the lack of trial evidence so far that introducing statins at age 20 will alter the rate at age 50 any more than introducing statins at, say, 45 years of age. Also for consideration is the lack of data on very long-term statin safety. Most vital is the need to avoid labelling young people with type 1 diabetes as being at high short-term risk of CVD if they are not, and to reassure when treatments are being based on long-term risks, or other considerations discussed above. To this end, we hope that the data and prediction tool we provide will be helpful to guideline committees and will facilitate conversations and decision making between clinicians and people with type 1 diabetes about when and why to start preventive therapies. For example, the model could be used in those below age 50 to detect those at high 10 year risk and ensure prevention strategies are maximised but also to reassure others with lower 10 year risk. We note that the Steno model is similarly useful for discrimination of risk as our own model, but it overestimated risk in our population.

### Ten year risk vs lifetime risk

The 2016 European Societies’ guideline explicitly recommends using 10 year risk, as we have done, rather than lifetime risk [7]. Lifetime estimates either make the incorrect assumption that current age-specific incidence rates will not change in the future or they make various untestable assumptions about how rates will change. Nonetheless, we provide the best available estimates of the cumulative probability of survival free of CVD by attained age derived from current age-specific rates as in Fig. 2. Note that when we show that the probability of being free of CVD by one’s mid-sixties is 50%, for a 20 year old today the true probability is very likely to be much higher than this in 40 years’ time if current calendar time trends of decreasing CVD rates in developed economies continue. We consider that it is better to be explicit about where uncertainties lie in presenting risks, and lifetime risk estimates will always carry substantial uncertainty.

### Study strengths and limitations

Strengths of our study are: (1) the complete coverage of the population and much larger dataset than for many studies of CVD in type 1 diabetes; (2) the provision of up-to-date absolute rates of events; (3) the use of statistical methods that avoid overfitting; and (4) the external validation in the Swedish NDR register. Limitations are that both our development and validation cohorts are European. Also, competing risks may mean that the absolute risk of CVD estimated by the model could be inflated where competing risks are common such as in the elderly, and use of the model in older individuals should take this into consideration. However, the envisaged utility of the model is more for risk prediction in younger individuals. As with all prediction models, prediction in different countries is optimised if there are available data to allow recalibration to reflect between-country differences in overall rates. We found only a slight recalibration was needed to reflect a slightly higher risk in the Swedish data, which is likely to reflect the earlier time period of coverage of the Swedish than Scottish data. Furthermore, different countries will need to substitute an equivalent socioeconomic indicator for the SIMD variable. For countries with no such indicator available we have provided an alternative model that does not use a socioeconomic indicator. Incidence and prevalence estimates will vary with the exact definition of CVD used. Our CVD outcome included both fatal and non-fatal CVD events, but to enhance specificity all the non-fatal events were hospital verified. By comparison, in the QRISK3 risk score derivation cohorts, more than 50% of all events were non-hospitalised events, including transient ischaemic attacks which are frequently false positive diagnoses [5, 22]. In contrast, the 2016 European Societies’ guideline Systematic Coronary Risk Evaluation (SCORE) risk charts focus only on fatal events on the basis that there is too much diagnostic variation between countries in non-fatal events [7]. Finally, we note that predictors from routinely collected clinical data will likely have a lower model performance than those collected in an idealised standardised setting. However, this will be a more realistic reflection of the performance in a real-world clinical setting.

### Conclusions

In conclusion, absolute rates of CVD in type 1 diabetes at younger ages are now lower than most international guidelines implicitly assume. Many people with type 1 diabetes below age 50 are not at high 10 year risk of CVD and should not be labelled as such. The contemporary data on risks of disease, and the provision of a risk prediction tool as presented here, could facilitate decision making on when and in whom to initiate CVD preventive therapies in type 1 diabetes. Such decisions may need to be made on a wider set of considerations than absolute 10 year risk alone.

## Supplementary Information

ESM (PDF 830 kb)

## Data Availability

NHS data governance rules do not permit us to secondarily share the data directly. However, bona fide researchers can apply to the Scottish Public Benefits and Privacy Protection Committee for access to these data. Code for the model script is available at https://github.com/diabepi/t1cvdrisk. This research was conducted with approval from the Public Benefit Privacy Protection Panel (PBPP ref. 1617- 0147). All datasets were de-identified before analysis.

## Abbreviations

NDR: National Diabetes Register
NICE: National Institute for Health and Care Excellence
SCI-Diabetes: Scottish Care Information – Diabetes
SIMD: Scottish Index of Multiple Deprivation
