# Supplementary material for: Development and validation of a cardiovascular risk prediction model in type 1 diabetes
Source: Diabetologia. 2021 Jun 9;64(9):2001–11. doi: 10.1007/s00125-021-05478-4 (PMC8382639; doi:10.1007/s00125-021-05478-4)
Supplement: Supplementary file 1 — (PDF 830 kb) [file 125_2021_5478_MOESM1_ESM.pdf]

ESM Methods

Study design and data sources

ICD & OPCS codes used for defining CVD

Prior CVD ICD codes used to exclude people who have had an event in a period of 10 years prior to the study start date

ICD10: I20, I21, I22, I23, I24, I25, I61, I63, I64, G45, I70.2, I70.8, I70.9, I73.9, E10.5, E11.5, E13.5, E14.5

ICD9: 410, 411, 412, 413, 414, 431, 433, 434, 435, 443.9, 440.2, 440.3, 440.4, 440.9, 250.7

Study CVD codes used to determine an event during the study period

ICD10: I20, I21, I22, I23, I24, I25, I61, I63, I64, G45, I70.2, I70.8, I70.9, I73.9, E10.5, E11.5, E13.5, E14.5

ICD9: 410, 411, 412, 413, 414, 431, 433, 434, 435, 443.9, 440.2, 440.3, 440.4, 440.9, 250.7

OPCS4: K40, K41, K42, K43, K44, K45, K46, K48, K49, K50, K75, L29, L30, L31.0, L31.1, L31.3, L31.4, L31.5, L31.6, L31.7, L31.8, L31.9, L33, L34, L35, X09.1, X09.2, X09.3, X09.4, X09.5

OPCS3: 304, 082.8, 871.2, 873

CHD related death during the study period

ICD10: I20, I21, I22, I23, I24, I25, I46

ICD9: 427.5, 410, 412, 413, 414

Candidate covariates

Clinician assigned diabetes type in SCI-Diabetes was accepted unless contradicted by available data on age at diagnosis and prescription history. Those who were assigned type 1 were reassigned as type 2 if they had a contradictory drug history and age at diagnosis >40 years. A prescription history that contradicts type 1 diabetes was more than 1-year of non-metformin oral diabetes drug use or more than a 1-year interval from diagnosis to insulin. This rule reassigned 10.8% of type 1 diabetes as type 2. Those who were initially assigned as type 2 were reassigned to type 1 only if they have insulin from diagnosis and an age of onset below age 30 years (0.8% were reassigned). Smoking was categorised according to the categories in the electronic health record front end to SCI-Diabetes; never/current/ex or unknown. Albuminuria was defined based on urinary albumin concentration in urines collected at routine clinic visits and categorised as normo-, micro- or macroalbuminuric according to the albumin/creatinine ratio (ACR) falling in the intervals 0–3.39 mg/mmol, 3.39–33.9 mg/mmol, or above 33.9 mg/mmol, based on two out of three most recent consecutive measurements. Retinopathy was defined based on grading of most recent retinal image at screening and categorised as none; non referable which is mild background; referable or at clinic which is pre-proliferative or worse retinopathy or maculopathy or prior referral to eye clinic because of retinopathy. The Scottish Index of Multiple Deprivation (SIMD) ranks geographic areas by a single index capturing seven domains of deprivation and is summarised in quintiles. Here,

for those with diabetes postcode of residence obtained from the CHI database was assigned to the SIMD quintile it belonged and was used as a measure of socioeconomic status.

Statistical methods

Model sensitivity checks

We confirmed that using models with backward selection or using multivariable fractional polynomials (using the mfp package in R) did not improve the AIC.

Special considerations on evaluating predictive performance form survival models

With a survival dataset reformatted as person-time intervals of fixed length, the predictive performance of the model can be evaluated by comparing "case" person-time intervals in which an event occurs with "noncase" person-time intervals in which no event occurs. The problem is that some of these person-time intervals are censored, either at the first event or at exit from other causes. Thus, the expected number of events in each person-time interval, calculated allowing for censoring at first event, does not rank individuals by risk. As Uno et al discuss [1], the classical C-statistic, which uses only the ranking of risk scores, cannot be used in this situation. Similar problems arise with calibration plots, as discussed by Crowson et al [2] and Austin et al [3].

The fundamental problem is that because person-time intervals of unequal length are not exchangeable, de Finetti's theorem does not apply; we cannot represent the case-noncase observations on these intervals as a mixture of independent and identically-distributed Bernoulli variables conditional on the risk scores If we ignore censoring and calculate the probability of an event before the end of a person-time interval of fixed length, the observations are exchangeable. This however inflates the total person-time of observation, so that the total expected events no longer equates to the total observed events; the "calibration in the large" cannot be evaluated.

Complicated methods have been proposed to overcome these problems [3] some of which for predictive performance have been implemented the SurvAUC package in R (https://cran.r-project.org/web/packages/survAUC/survAUC.pdf accessed 06 Dec 2020). A much simpler approach is to reformat the dataset with many short person-time intervals. In the limit of infinitesimally short person-time intervals, the Bernoulli and Poisson likelihoods are equivalent and censoring within person-time intervals can be ignored. We can then use standard methods to evaluate the predictive performance and calibration of a binary classifier, as in a case-control study. Thus, for the purposes of assessing calibration and predictive performance both in the concatenated cross-validation test dataset in Scotland and in the Swedish dataset the one year person time intervals were subdivided into 28-day intervals as no further change in estimated calibration occurred beyond this (see http://www.homepages.ed.ac.uk/pmckeigu/stats/survival.html accessed 6 Dec 2020) for further theoretical underpinning of this approach and a worked example.

Re-calibration of the model in Sweden

To re-calibrate the model for Sweden, the predicted rate was adjusted on the log scale as follows; If P is the average rate in one person year in the target population, and Q is the average rate in one person year using the model in the target population, the di˙erence on the log scale gamma is calculated as gamma = log(P) - log(Q). For each individual in the Swedish dataset, the risk obtained from the model was then adjusted by transforming it to the log scale, adding gamma, and transforming it back to the risk scale.

ESM Results

Scottish model implementation

For a single individual the probability of an event within the next T years, assuming no censoring from other causes, is

$$1 - \prod_{t=1}^{T} (1 - \exp \mu_t)$$

where

$$\mu_t = \exp\left(\alpha + \boldsymbol{X_t}\boldsymbol{\beta}\right)$$

where α is the intercept parameter, $\boldsymbol{X_t}$ is a matrix of covariates with one row for each of the T time intervals and $\boldsymbol{\beta}$ is the vector of coeÿcien ts in the Poisson regression model. Covariates, intercept and coeÿcien ts are provided in ESM table 9.

ESM references

1. Uno H, Cai T, Pencina MJ, D'Agostino RB et al. On the C-statistics for evaluating overall adequacy of risk prediction procedures with censored survival data. Stat Med. 2011 May 10;30(10):1105-17. doi: 10.1002/sim.4154. Epub 2011 Jan 13. PMID: 21484848; PMCID: PMC3079915.
2. Crowson CS, Atkinson EJ, Therneau TM. Assessing calibration of prognostic risk scores. Stat Methods Med Res. 2016 Aug;25(4):1692-706. doi: 10.1177/0962280213497434. Epub 2013 Jul 30. Erratum in: Stat Methods Med Res. 2017 Aug;26(4):1992-1993. PMID: 23907781; PMCID: PMC3933449.
3. Austin PC, Harrell FE Jr, van Klaveren D. Graphical calibration curves and the integrated calibration index (ICI) for survival models. Stat Med. 2020 Sep 20;39(21):2714-2742. doi: 10.1002/sim.8570. Epub 2020 Jun 16. PMID: 32548928; PMCID: PMC7497089.

ESM Table 2: Scottish dataset missingness

	Missingness(%)
Age at entry (years)	0.00
Sex	0.00
Diabetes duration (years)	0.00
Deprivation index	9.64
BMI (kg/m^2)	6.04
eGFR (ml min^{-1} 1.73m^{-2})	13.65
Mean HbA$_{1c}$ (last 3 years)(mmol/mol)	4.34
HbA$_{1c}$ (mmol/mol)	5.26
Systolic BP (mmHg)	4.31
Diastolic BP (mmHg)	4.30
Weight (kg)	4.43
Height (meters)	6.06
Total cholesterol (mmol/l)	11.07
HDL cholesterol (mmol/l)	21.86
Albuminuric grade	35.88
Retinopathy grading	18.05
Tobacco smoking	1.38
Exercise	66.15
Treated for hypertension	0.00
Treated for dyslipidaemia	0.00
Ever atrial fibrillation	0.00

If there was no history of treatment for hypertension, or history of treatmemt of dyslipidaemia or history of atrial fibrillation, the variable was encoded as 0, so by definition there is zero missingness for continuous values

ESM Table 1: Scottish cohort characteristics at study entry

	No CVD	CVD	P-Value	Total
**Total included**	24737(89.9%)	2790(10.1%)		27527
Total Follow-up (years)	185577(93.0)	13975(7.0)		199552
**Sociodemographic**				
Sex				
Male	14041(56.8)	1658(59.4)		15699(57.0)
Female	10696(43.2)	1132(40.6)	0.004	11828(43.0)
Age at entry (years)	33(22,44)	49(41,59)	<0.001	35(23,46)
Diabetes duration (years)	12(4,21)	24(15,34)	<0.001	13(5,23)
Age at diabetes diag (years)	19(11,29)	25(15,34)	<0.001	19(11,30)
Follow up (years)	10(5,10)	5(3,8)	<0.001	10(4,10)
Year diagnosed (year)	1997(1987,2006)	1984(1974,1993)	<0.001	1996(1985,2005)
Deprivation index				
Quintile 1 (most deprived)	4620(18.7)	644(23.1)	<0.001	5264(19.1)
Quintile 2	4726(19.1)	610(21.9)	0.121	5336(19.4)
Quintile 3	4534(18.3)	487(17.5)	<0.001	5021(18.2)
Quintile 4	4415(17.8)	394(14.1)	<0.001	4809(17.5)
Quintile 5 (least deprived)	4214(17.0)	299(10.7)	<0.001	4513(16.4)
Unknown	2228(9.0)	356(12.8)	<0.001	2584(9.4)
**Other clinical measures**				
HbA_{1c} (mmol/mol)	72(61,85)	77(65,91)	<0.001	72(61,86)
HbA_{1c} (%)	8.69(7.73,9.93)	9.20(8.10,10.48)	<0.001	8.74(7.73,10.02)
BMI (kg/m^2)	25(23,29)	27(24,30)	<0.001	26(23,29)
Height (meters)	1.71(1.64,1.78)	1.70(1.62,1.77)	<0.001	1.71(1.64,1.78)
Weight (kg)	74(65,85)	78(67,90)	<0.001	75(65,86)
Systolic BP (mmHg)	126(117,136)	134(123,144)	<0.001	127(118,138)
Diastolic BP (mmHg)	75(69,80)	75(69,81)	0.411	75(69,80)
Total cholesterol / HDL ratio (mmol/l)	3.18(2.64,3.85)	3.20(2.59,3.96)	<0.001	3.18(2.63,3.86)
eGFR (ml min^{-1} 1.73m^{-2})	101(86,116)	83(68,98)	<0.001	99(84,115)
Albuminuric status				
Normal	13095(52.9)	1215(43.5)		14310(52.0)
Micro	2268(9.2)	491(17.6)	<0.001	2759(10.0)
Macro	311(1.3)	165(5.9)	<0.001	476(1.7)
Unknown	9063(36.6)	919(32.9)	<0.001	9982(36.3)
Retinopathy				
None	9709(39.2)	454(16.3)		10163(36.9)
Non referable	6776(27.4)	751(26.9)	<0.001	7527(27.3)
Referable / eye clinic	3687(14.9)	1110(39.8)	<0.001	4797(17.4)
Unknown	4565(18.5)	475(17.0)	<0.001	5040(18.3)
Tobacco smoking status				
Never smoked	11778(47.6)	754(27.0)		12532(45.5)
Ever smoked	12575(50.8)	2019(72.4)	<0.001	14594(53.0)
Unknown	384(1.6)	17(0.6)	<0.001	401(1.5)
**Comorbidities**				
No. with atrial fibrillation	25(0.1)	20(0.7)	<0.001	45(0.2)
No. treated for dyslipidaemia	7570(30.6)	1965(70.4)	<0.001	9535(34.6)
No. treated for hypertension	6163(24.9)	1798(64.4)	<0.001	7961(28.9)

Data are shown in N(%) for categorical values and median interquartile range for continuous values. Cases relate to CVD events that occurred during follow-up

ESM Table 3: Associations of each risk factor considered in the modelling process with incident first CVD event in the Scottish data adjusted for age, sex and diabetes duration

Predictor	IRR (95% CI)	P-Value
Age at entry (years)	1.163 (1.146, 1.180)	<0.001
Current age (years)	1.046 (1.043, 1.050)	<0.001
Year diagnosed (year)	0.942 (0.908, 0.978)	0.002
Sex		
Male	1 (reference)	
Female	0.831 (0.770, 0.896)	<0.001
Diabetes duration (years at entry)	1.023 (1.019, 1.026)	<0.001
Deprivation index		
Quintile 1 (most deprived)	1 (reference)	
Quintile 2	0.825 (0.739, 0.919)	0.001
Quintile 3	0.623 (0.554, 0.700)	<0.001
Quintile 4	0.524 (0.463, 0.593)	<0.001
Quintile 5 (least deprived)	0.389 (0.340, 0.445)	<0.001
HbA_{1c} (mmol/mol)	1.030 (1.028, 1.032)	<0.001
Mean HbA_{1c} (last 3 years)(mmol/mol)	1.031 (1.029, 1.033)	<0.001
BMI (kg/m^2)	1.015 (1.008, 1.023)	<0.001
Height (meters)	0.147 (0.086, 0.253)	<0.001
Weight (kg)	1.001 (0.998, 1.004)	0.429
Systolic BP (mmHg)	1.007 (1.004, 1.009)	<0.001
Diastolic BP (mmHg)	1.006 (1.002, 1.010)	0.005
Total cholesterol / HDL ratio (mmol/l)	1.049 (1.041, 1.056)	<0.001
eGFR (ml min^{-1} $1.73m^{-2}$)	0.988 (0.985, 0.990)	<0.001
Albuminuric grade		
Normal	1 (reference)	
Micro	2.118 (1.917, 2.339)	<0.001
Macro	4.978 (4.274, 5.797)	<0.001
Retinopathy grading		
None	1 (reference)	
Non referable	1.203 (1.074, 1.347)	0.001
Referable or eye clinic	2.182 (1.956, 2.435)	<0.001
Tobacco smoking		
Never smoked	1 (reference)	
Ever smoked	1.746 (1.605, 1.899)	<0.001
Treated for hypertension	1.957 (1.794, 2.135)	<0.001
Treated for dyslipidaemia	1.673 (1.525, 1.836)	<0.001
Ever atrial fibrillation	2.465 (1.585, 3.835)	<0.001

ESM Table 4: Swedish cohort characteristics at study entry

	No CVD	CVD	P-Value	Total
**Total included**	29921(90.17)	3262(9.83)		33183
Total Follow-up (years)	237649(94)	15548(6)		253197
**Sociodemographic**				
Sex				
Male	16319(54.54)	1801(55.21)		18120(54.61)
Female	13602(45.46)	1461(44.79)	0.681	15063(45.39)
Age at study entry (years)	30.0(21.5,41.1)	51.9(42.7,60.1)	<0.001	31.9(22.2,44.3)
Diabetes duration (years)	15.4(7.6,25.9)	35.7(26.3,45.0)	<0.001	16.9(8.4,28.8)
Age at diag (years)	14.4(9.2,21.5)	15.2(9.6,22.9)	0.086	14.4(9.2,21.7)
Follow up (years)	9.0(5.1,11.0)	4.4(2.0,7.3)	<0.001	8.6(4.5,10.9)
Year diagnosed (year)	1990(1978,1999)	1968(1958,1977)	<0.001	1988(1975,1998)
Deprivation index				
Quantile 1 (most deprived)	6695(22.38)	266(8.15)		6961(20.98)
Quantile 2	5565(18.60)	846(25.94)	<0.001	6411(19.32)
Quantile 3	5583(18.66)	824(25.26)	<0.001	6407(19.31)
Quantile 4	5989(20.02)	701(21.49)	<0.001	6690(20.16)
Quantile 5 (least deprived)	6089(20.35)	625(19.16)	<0.001	6714(20.23)
**Other clinical measures**				
HbA_{1c} (mmol/mol)	64(54,73)	68(59,78)	<0.001	64(55,74)
HbA_{1c} (%)	8.0 (7.1, 8.8)	8.4 (7.5, 9.2)	<0.001	8.0 (7.2, 8.9)
BMI (kg/m^2)	24.5(22.3,27.2)	25.1(22.6,27.9)	<0.001	24.6(22.3,27.3)
Height(M)	1.73(1.66,1.80)	1.72(1.64,1.79)	<0.001	1.73(1.66,1.80)
Systolic BP (mmHg)	120(114,130)	140(125,150)	<0.001	122(115,135)
Diastolic BP (mmHg)	72(68,80)	75(70,80)	<0.001	72(69,80)
Total Cholesterol / HDL ratio (mmol/l)	3.1(2.5,3.7)	3.2(2.6,4.1)	<0.001	3.1(2.5,3.8)
eGFR (ml min^{-1} 1.73m^{-2})	95.1(78.0,113.9)	74.3(56.1,92.3)	<0.001	93.2(75.7,112.4)
Albuminuric Status				
Normo	24963(83.43)	1771(54.29)		26734(80.57)
Micro	3284(10.98)	745(22.84)	<0.001	4029(12.14)
Macro	1674(5.59)	746(22.87)	<0.001	2420(7.29)
Retinopathy Status				
None	16182(54.08)	843(25.84)		17025(51.31)
Retinopathy	13739(45.92)	2419(74.16)	<0.001	16158(48.69)
Tobacco smoking status				
Ever Smokers	3555(11.88)	620(19.01)	<0.001	4175(12.58)
**Comorbidities**				
No. with atrial fibrillation	30(0.10)	15(0.46)	<0.001	45(0.14)
No. prescribed statins	3049(10.19)	1124(34.46)	<0.001	4173(12.58)
No. prescribed anti-hypertensive	5192(17.35)	1867(57.23)	<0.001	7059(21.27)

Data are shown in N(%) for categorical values and median interquartile range for continuous values. Cases relate to CVD events that occurred during follow-up

ESM Table 5 Swedish dataset missingness

	Missingness(%)
Age at entry (years)	0.00
Sex	0.00
Diabetes duration (years)	0.00
Deprivation index	0.10
BMI (kg/m^2)	13.60
eGFR (ml min^{-1} 1.73m^{-2})	25.90
HbA_{1c} (mmol/mol)	4.40
Systolic BP (mmHg)	6.70
Diastolic BP (mmHg)	6.70
Weight (kg)	8.30
Height (meters)	11.00
Total cholesterol (mmol/l)	32.00
HDL cholesterol (mmol/l)	36.70
Albuminuric grade	17.60
Retinopathy grading	61.10
Tobacco smoking	0.00
Treated for hypertension	0.00
Treated for dyslipidaemia	0.00
Ever atrial fibrillation	0.00

If there was no history of treatment for hypertension, or history of treatmemt of dyslipidaemia or history of atrial fibrillation, the variable was encoded as 0, so by definition there is zero missingnessmedian interquartile range for continuous values

ESM Table 6: Associations of each risk factor considered in the modelling process with incident first CVD event in the Swedish data adjusted for age, sex and diabetes duration

Predictor	IRR (95% CI)	P-Value
Age at study entry (years)	1.064 (1.050, 1.078)	<0.001
Current age (years)	1.054 (1.048, 1.060)	<0.001
Year diagnosed (year)	0.933 (0.921, 0.945)	<0.001
Sex		
Male	1 (reference)	
Female	0.812 (0.747, 0.883)	<0.001
Diabetes duration (years at entry)	1.029 (1.024, 1.035)	<0.001
Deprivation index		
Quintile 1 (most deprived)		
Quintile 2	1.377 (1.167, 1.625)	<0.001
Quintile 3	1.283 (1.088, 1.513)	0.003
Quintile 4	0.986 (0.832, 1.169)	0.873
Quintile 5 (least deprived)	0.774 (0.650, 0.923)	0.004
HbA_{1c} (mmol/mol)	1.035 (1.032, 1.038)	<0.001
BMI (kg/m^2)	1.026 (1.015, 1.036)	<0.001
Height(M)	0.219 (0.118, 0.407)	<0.001
Weight (kg)	1.004 (1.001, 1.008)	0.012
Systolic BP (mmHg)	1.017 (1.015, 1.020)	<0.001
Diastolic BP (mmHg)	1.020 (1.016, 1.025)	<0.001
Total Cholesterol / HDL ratio (mmol/l)	1.217 (1.205, 1.229)	<0.001
eGFR (ml min^{-1} $1.73m^{-2}$)	0.990 (0.989, 0.992)	<0.001
Almuninuric grade		
Normal	1 (reference)	
Micro	1.908 (1.719, 2.118)	<0.001
Macro	3.768 (3.400, 4.176)	<0.001
Retinopathy		
None	1 (reference)	
Mild/Moderate/Refereable	1.361 (1.233, 1.503)	<0.001
Tobacco smoking		
Never smoked	1 (reference)	
Ever Smoker	1.856 (1.669, 2.064)	<0.001
Treated for hypertension	1.974 (1.699, 2.295)	<0.001
Treated for dyslipidaemia	1.866 (1.545, 2.253)	<0.001
Ever atrial fibrilation	1.240 (0.665, 2.314)	0.499

ESM Table 7: Predictive performance of the QRISK3 and the Steno CVD Type 1 Engine in the Scottish population

Model	C-Statistic (95% CI)	Observed events	Events predicted	Ratio
Steno	0.82 (0.81 ,0.83)	2790	3557	1.27
QRISK3	0.75 (0.74 ,0.76)	2654	1917	0.72

Observed events are lower for QRISK, as the model has an age limit of 25-84

ESM Table 8: Percentage eligible for statin therapy under NICE guideline and percentage with a 10 year risk of at least 5% and at least 10%

Age group	Eligible for Statin	CVD Risk $\geq$ 5%	CVD Risk $\geq$ 10%
20-24	81.35	4.29	1.19
25-29	89.13	10.91	3.49
30-34	92.47	24.55	9.11
35-39	95.14	47.03	20.85
40-44	100.00	69.92	37.10
45-49	100.00	85.13	56.80
50-54	100.00	95.15	75.23
55-59	100.00	98.56	85.55
60-64	100.00	99.50	95.23
65-99	100.00	100.00	98.65
70-74	100.00	100.00	100.00
75-79	100.00	100.00	100.00
80-84	100.00	100.00	100.00
85-89	100.00	100.00	100.00

Risks were increased by 25% for individuals receiving statin therapy at study entry

ESM Table 9: Predictor variables and coefficients for risk prediction of CVD in type 1 diabetes excluding deprivation index

Predictor	Coefficient
(Intercept)	-3.64810112
Current age (years)	0.3271865
Current age^2 (years^2)	-0.00403131
Current age^3 (years^3)	2.08×10^{-05}
Sex male	0.00000000
Sex female	-0.77934644
Diabetes duration (years)	0.01943773
HbA_{1c} (mmol/mol)	0.01143295
Mean HbA_{1c} (last 3 years)(mmol/mol)	0.02781042
log BMI (kg/m^2)	-2.01243842
Height (meters)	-3.18108541
Weight (kg)	0.00741941
Systolic BP (mmHg)	0.00381897
log Total cholesterol / HDL ratio (mmol/l)	0.49215468
log eGFR (ml min^{-1} 1.73m^{-2})	-0.32126549
Albuminuric grade normal	0.00000000
Albuminuric grade micro	0.36310623
Albuminuric grade macro	0.94826311
Retinopathy none	0.00000000
Retinopathy non referable	0.10606169
Retinopathy referable or eye clinic	0.41224979
Never smoked	0.00000000
Tobacco smoking	0.38809494
Treated for hypertension	0.31887555
Treated for dyslipidaemia	0.14728126
Ever atrial fibrillation	0.70356665
Interaction: Sex * log Total cholesterol / HDL ratio	0.48736885
Interaction: Age * Weight	0.0002678
Interaction: Age * 3 year mean HbA_{1c}	-0.00028851

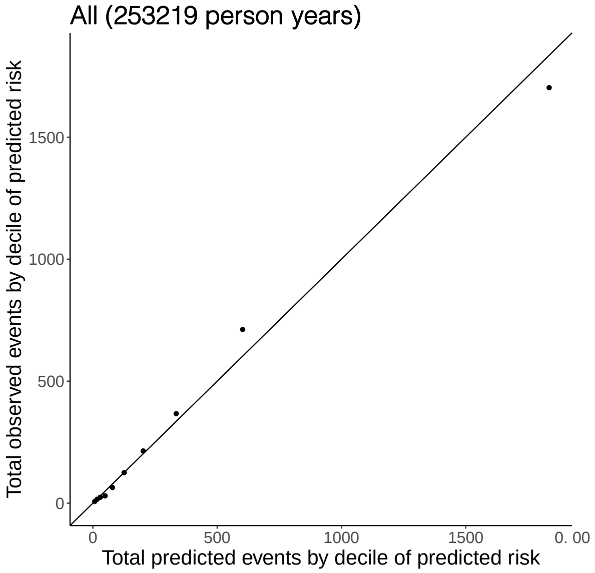

ESM Fig 1: Calibration of model in the Swedish validation dataset
